# A New State Medical Service Plan

**Published:** 1919-02-01

**Authors:** 


### THE FUTURE OF THE MEDICAL PROFESSION.

### A New State Medical Service Plan.

In the *British Medical Journal* of January 18, 1918, Dr. P. R. Cooper suggests a plan for a State Medical Service on the principle of payment for work done. He begins his article with the statement, "The omens at present point to the early establishment of some kind of State Medical Service." This has been pointed out often in the pages of The Hospital.

Many straws show that the wind blows that way. This being so, Dr. Cooper's suggestion is valuable, because it is of the utmost importance to the medical profession that every possible plan of service should be examined and discussed. Peradventure from the discussion may emerge a wise and statesmanlike scheme. Dr. Cooper has hit upon one valid objection, though not the only one, in the last paragraph of his article. The coupon scheme "might be abused by some medical men," he says. It is perfectly certain that it would be grossly abused by some. The black sheep of the profession would make a good thing out of it, and be readily helped to do so by their patients, for, unfortunately, the mass of the community is not yet educated up to the point of being scrupulously honest where State money is in question. Men and women alike do not feel their conscience greatly oppressed by evasion of income tax, dog tax, game licences, and many other lawful dues. Every man is a smuggler on occasion. It is notorious and has been very evident to all who have served in this war that men and women are careless of Government property, and misuse and waste it in a way that they never waste their own or private persons' property. A still greater number of medical men will not be able to resist the temptation to get just one or two more fees than they would have felt it wise to ask if the charge had to be put in a bill to a private patient, and a great many patients will indulge themselves in a pleasant chat with their doctor, when it only costs a coupon, who would have foregone the pleasure had they had to pay a fee. The result would certainly be greater expenditure on "doctoring" than need be, and the less conscientious the doctor the better off he would be. Checks are hard to devise, not easy, as Dr. Cooper imagines. All who have followed legal cases for personal damages against railway companies and such corporations know well how greatly the bias of juries is for the individual as against the corporate body. The State would be in the position of a corporation in every dispute.

But, granting Dr. Cooper's scheme to be satisfactory as a way of helping people to pay doctors' bills, and a way of enabling doctors easily to collect just debts, it fails as a scheme for a medical service because it does not begin to suggest any scheme for doing what a medical service to be worth having has got to do. A medical service ought to be the tool to the hand of a wide-minded far-visioned Ministry of Health; its instrument to prevent disease, to treat disease, to cure disease, to improve medical education, to deal with those of feeble mind, and to improve the physique of the British race. It is definitely set forth in the Ministry of Health Act that these things the new Minister shall do, and it is because he has to do these things and cannot do them without a State Medical Service that many minds are bent on plans for such a service. Dr. Cooper's suggestion may be valuable as showing a way of distributing pay to State medical officers, or a way of estimating the work each does for his pay, but to hand out books of coupons to prospective sick persons and let the doctor collect his bills by passing a collection of coupons to an insurance company and calling on Parliament to make good any deficit is not making a State Medical Service.

